# Deciding Thrombolysis in AIS Based on Automated versus on WhatsApp Interpreted ASPECTS, a Reliability and Cost-Effectiveness Analysis in Developing System of Care

**DOI:** 10.3389/fneur.2020.00333

**Published:** 2020-05-19

**Authors:** Ossama Yassin Mansour, Ismail Ramadan, Ashraf Abdo, Mohamed Hamdi, Hany Eldeeb, Hazem Marouf, Doaa Elsalamawy, Amr Elfatatry, Abdelaziz Elnekidy, M. Ihab Reda

**Affiliations:** ^1^Alexandria Stroke and Neurointerventional Services, Alexandria University, Alexandria, Egypt; ^2^Neurology Department, Alexandria University, Alexandria, Egypt; ^3^Neuroradiology Department, Alexandria University, Alexandria, Egypt

**Keywords:** acute ischemic stroke, thrombolysis, automated ASPECTS, mRS, reliability, cost-effectiveness analysis

## Abstract

**Background:** Automated ASPECTS has the potential of reducing interobserver variability in the determination of early ischemic changes. We aimed to assess the performance of an automated ASPECTS vs. ASPECTS interpreted for sent CT images on WhatsApp and to correlate these results with the outcome.

**Materials and Methods:** Patients with anterior circulation stroke who had baseline NCCT and underwent successful IV-thrombolysis were included. NCCT-ASPECTS was assessed by two neuroradiologists, and discrepancies were resolved by agreement. Two groups of patients were included; group 1, where treatment was decided after an automated ASPECTS interpretation that was provided by RAPID software, and group 2, where patients received IV-tPA after an assessment of CT images sent on WhatsApp.

**Results:** A total of 122 patients were included: 36 in group 1 and 86 in group 2. In group 2, the interobserver agreement for NCCT ASPECTS was moderate (κ = 0.36), as was the dichotomized data (κ = 0.44). IOA, however, improved (to κ = 0.57 and κ = 0.64) when the same CT images were interpreted on a workstation. In group 1, Automated ASPECTS showed excellent agreement (κ = 0.80) with agreement reads for workstation images. There were significantly (*P* < 0.001) increased odds of functional independence and fewer hemorrhagic complications with thrombolyzed patients in group 1.

**Conclusions:** Automated ASPECTS provided by the RAPID@IschemaView ASPECTS performs at a level equal to the agreement read of expert neuroradiologists, and this performance was severely degraded when WhatsApp captured CT images used for ASPECTS assessment. In our study, we found that automated ASPECTS might predict outcomes after IV thrombolysis.

## Introduction

ASPECTS was introduced in the year 2000 to assess the early ischemic changes in CT scans. It is a 10-point scoring system, with one point deducted for signs of early ischemic change in each defined region of the MCA territory (1).

CT-ASPECTS has been shown to predict the functional outcome and symptomatic intracranial hemorrhage after thrombolytic treatment (1, 2).

Consequently, the ASPECTS assessment has been increasingly incorporated into treatment decision making and has been used in several randomized clinical trials for endovascular treatment decision making (3, 4).

The major drawback of the ASPECTS evaluation is its modest interobserver agreement (IOA) and reproducibility. Early ischemic changes are often difficult to detect on NCCT, with low interobserver agreement for presence and extent (5–7).

Transmitting CT scans as instant messages captured and sent using smartphones has been shown to be highly reliable in neurosurgical emergencies (8).

Although scientific studies on the use of WhatsApp Messenger remain scarce in medical literature, increasing numbers of health professionals have adopted it as a communication interface and for the exchange of multimedia (9, 10). A debate is currently ongoing on the subject of its effect on transmitted image quality in the conversion from analog to digital formats to the degree of whether it is able to identify sufficient detail for an adequate diagnosis and initial treatment with better efficacy than other modalities used for the same purposes (11).

In acute ischemic stroke settings, both time and detail are imperative to confounding the incorporated role of ASPECTS in treatment decision-making process.

To our knowledge, this is the first study to evaluate the reliability of interpreting ASPECTS from three settings—source images, captured images on WhatsApp, and automated ASPECTS obtained from a software-based analysis (RAPID ASPECTS®) by iSchemaView (Menlo Park, USA www.ischemaview.com)—and to show the possible impact on the outcome after thrombolysis.

## Materials and Methods

### Patient Selection

This study was approved by the local institutional review board (Alexandria University System, Alexandria). We retrospectively reviewed consecutive patients with AIS who presented to the primary stroke unit, which is one our institution-affiliated facilities for stroke care in the Alexandria stroke network (www.egyptianstroke.net). Between January 2018 and December 2019, 176 AIS patients who met the following inclusion criteria received IV-tPA: (1) time from symptom onset <4.5 h; (2) anterior circulation ischemic stroke; (3) baseline NCCT; (4) no contraindication for IV-tPA; and (5) ASPECT score ≥ 6 (ischemic changes ≤ 1/3 of MCA territory). There were several exclusion criteria: (1) intracranial hemorrhage; (2) pre-existing cerebral defects within the probable current ischemic area that could not reliably be distinguished from acute ischemic changes; and (3) severe motion or other artifacts impeding CT interpretation. To expedite stroke workflow, the thrombolysis decision is remotely taken by a stroke consultant through a closed WhatsApp group where clinical and laboratory data of the patients as well as NCCT images are uploaded by a stroke residency where interpretation of NCCT is carried out on the WhatsApp group by the neuroradiologist on duty. Other stroke neurologists, neurosurgeons, and ICU physicians all are within the same chat group and are notified by the decision. As of 2019, RAPID software (a computer-based automated scoring to assess early signs of brain ischemia) has been deployed in our institution to help develop a faster and more accurate stroke workflow as a part of developing the stroke service to include all reperfusion therapies for AIS. During our study period, we could identify two groups of patients: group 1 consisted of 36 AIS patients receiving the IV-tPA based on the automated ASPECTS processed by the RAPID system, while group 2 consisted of 86 AIS patients receiving treatment based on the decision made using the NCCT images sent via the WhatsApp group.

All clinical data for both groups of patients included the patients' age, sex, baseline NIHSS scores, the time from stroke onset/last well-known, door to need time, time from CT to needle, data on receiving intravenous tissue plasminogen activator, and 90-day mRS when available. mRS scores <3 were used to indicating a functional independence outcome.

### Image Acquisition

CT image acquisition was performed using a Brilliance 64-slice CT scanner (Philips Healthcare, Netherland). Helical NCCT (120 kV, 100–350 auto-mA) was performed using a 5-mm section thickness from the foramen magnum through the vertex.

### WhatsApp Data Transmission

Clear regulations and standardization of medical data sharing are lacking, and the use of Whatsapp(TM) remains a “gray area.” We tried to reduce variation in the image quality transmitted via WhatsApp, where all transferred NECT images were captured by one universal smartphone—the “resident's phone” (Iphone 8 plus; 8MP camera). The transmitted images were either made up of a full range of series with customized slice thickness (1mm) generated by CT workstation software for printing or a video clip spanning the entire CT study. However, other factors, such as the “camera view angle” and “image light intensity,” that could affect image quality could not be avoided in this study, and this might downgrade the efficiency of the WhatsApp method for transmitting CT images when deciding on AIS treatment compared to a decision based on transferred automated dicom files through dedicated software.

On the other hand, in WhatsApp-transmitted images, trying to reduce concerns regarding the identification of patients from non-anonymized shared data was considered in the current study where all data were transmitted to a closed group on WhatsApp that included only physicians who are involved in the treatment workflow of the patient. However, other privacy concerns could not be avoided, such as the fact that images sent through the app will be immediately downloaded into the recipient's smartphone photo library unless that setting is manually switched off. All messages are stored on a server in the US, which means they are not compliant with UK data protection legislation and the General Data Protection Regulation (GDPR). We did not anonymize the process of transmitting the images for the sake of the time factor, which is crucial in AIS treatment.

### Image Analysis (Figure 1)

Two neuroradiologists years with more than 10 years' experience independently reviewed all baseline NCCTs and assigned an ASPECTS using a 10-point scale (1). Discrepancies between two readers were resolved using an agreement read in a separate reading session.

**Figure 1:**
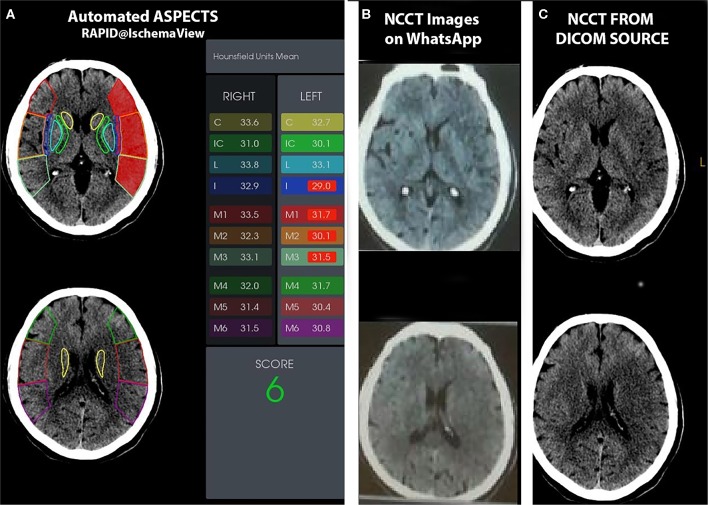
A 70-year-old man who presented with right hemiparesis, dysphasia with right UMN facial weakness, and an NIHSS score of 16. He underwent IV thrombolysis with a door to needle time of 40 min. Automated ASPECTS **(A)**, Axial NCCT images uploaded to the WhatsApp group **(B)**, and axial NCCT images processed by NR on workstation **(C)** are shown. For the two human readers, one scored 6 and the other 7 (agreement ASPECTS, 6). **(B)** Automated software assigned an ASPECTS of 6.

In addition, an automated software-based analysis RAPID ASPECTS® by iSchemaView (Menlo Park, USA). Axial isotropic sequences from the NCCT for each patient were uploaded to the software, and the automated ASPECTS was calculated without human interaction.

For both group of patients, the neuroradiologists and automated ASPECTS were then exported to an IBM SPSS software V23 spreadsheet (IBM corporation; Giza, Egypt) for analysis.

Finally, we used a cost-effectiveness analysis to compare the use of the automated ASPECTS interpretation (RAPID ischemaView) and the traditional ASPECTS interpretation of the sent NCCT images on the WhatsApp group.

A CEA decision tree model was created by TreeAge Pro 2019 (TreeAge Software, inc.) to evaluate the cost-utility analysis for both traditional and automated interpretation of ASPECTS CT., where we assumed that 90 days of follow-up could be one of the three possibilities, using mRS to reflect clinical outcome after IV-tPA: either 1-functional independence; 0–2 mRS; 2- functional dependence; 3–5 mRS; or 6 mRS - death. For cost utility, we refer you to Table 1.

**Table 1:** Characteristics of 122 patients who received IV-tPA in both groups.

	**WhatsApp (*n* = 86) group 2**	**RAPIDsoftware (*n* = 36) group 1**	***P***
Age (Mean)	58.2 ± 9.4	60.9 ± 10.5	0.172
Door to needle (min)	52.3 ± 16.0	36.8 ± 11.8	0.001
Male	46 (53.4%)	13 (36.3%)	0.080
HTN	36 (41.9%)	20 (55.6%)	0.166
DM	32 (37.2%)	12 (33.3%)	0.684
Smoker	11(12.8%)	9 (25%)	0.097
Dyslipidemia	9 (10.5%)	4 (11.1%)	0.916
AF/arrythmias	14 (16.3%)	9 (25%)	0.261
IHD	30 (34.9%)	12 (33.3%)	0.869
Hgic transformation	18 (2 0.9%)	2 (5.6%)	0.036
Dyas90mRS (0–3)	35 (40.7%)	23 (63.9%)	0.030
Dichotomized ASPECTS ≥6	34 (94.4%)	75 (87.2%)	0.3

### Statistical Analysis

Clinical and demographic data were presented as mean (SD) or median (interquartile range) as appropriate. ASPECTS values were presented as median (interquartile range). Comparison of ASPECTS was performed using both the raw/original scores and dichotomized ASPECTS using ≥6 and <6 as a cutoff. The interobserver agreement between two neuroradiologists was performed using a weighted κ test with a calculation of the 95% CI. Agreement and correlation among neuroradiologist agreement reads and automated ASPECTS were performed using the intraclass correlation coefficient with 95% CI.

Clinical and imaging variables and functional outcome (using a 90-day mRS >2 as a cutoff) were compared between both groups using a combination of *t*-tests and χ^2^ tests as appropriate.

## Results

### Demographic and Clinical Data

A total of 122 patients were included (59 men, 63 women; mean age, 59 ± 9.8 years). The mean of the door to needle time was 47.8 ± 16.5 min. The median and interquartile range (IQR) of the NIHSS were 11 and 8–14. Stroke etiology was cardioembolic in 27.9% of cases. A total of 58/122 patients (47.5%) achieved mRS <3 at 90-days follow-up. The median and (IQR) of the final agreement ASPECTS (inferred from source Dicom images) were 8 and 6–9.

Table 1 shows the basic characteristics of AIS patients in both groups of patients. A higher rate of hemorrhagic complications was seen in thrombolysis decided based on the NCCT shared on WhatsApp (20 vs. 5.5%, *P* = 0.036). By dichotomizing the 90-day outcome by using mRS < 3 as a cutoff value, indicating functional independence, a higher incidence of getting functional independence was observed in thrombolysis based on automated ASPECTS CT interpretation (40.7 vs. 63.9%, *P* = 0.022). Additionally, a 14-min reduction in the DTN time was observed in the group of AIS patients with automated ASPECTS interpretation (median of 50 and 36 min in group 1 and group 2, respectively).

### Human Interpretation

In group 2, where the NCCT was delivered via WhatsApp (*n* = 86), an NCCT file that was uploaded to the WhatsApp group was read by two neuroradiologists to interpret the ASPECTS. Similarly, both were involved blindly (without knowing localizing information) to rate the ASPECTS of the NCCT dicom source images on the workstation for each patient.

The median for ASPECTS rated from CT images sent on WhatsApp was 7 (IQR, 6–7) for reader 1 and 7 (IQR, 6–8) for reader 2. The interobserver agreement was fair with κ = 0.36 (95% CI, 0.02–0.58). For the dichotomized ASPECTS (ASPECTS ≥6 or <6), the interobserver agreement was improved to κ = 0.44 (95% CI, 0.14–0.64).

When readers were involved in interpreting ASPECTS from the source of the images on the workstation for the same patients, the median for ASPECTS was 7 (IQR, 5–7) for reader 1 and 7 (IQR, 6–8) for reader 2, and the IOA was improved to moderate with κ = 0.57 (95% CI, 0.33–0.72). Similarly, for the dichotomized ASPECTS (ASPECTS ≥6 or <6), IOA was improved with κ = 0.69 (95% CI, 0.51–0.79). In the dichotomized agreement read, a total of 75 patients had ASPECTS ≥6 and 11 patients had ASPECTS <6.

### Automated ASPECTS of NCCT

In group 2, where 36 patients received IV-tPA based on automated ASPECTS generated by RAPID^@^ IschemaView software, the median was 7 (IQR, 6–8) for automated ASPECTS. The source Dicom images were blindly evaluated by both readers in an agreement session in which, for the final agreement read, the median for ASPECTS was 7 (IQR, 7–9).

By dichotomizing Automated ASPECTS, a total of 35 patients had eASPECTS ≥6, while 1 had eASPECTS <6.

Comparing the ASPECTS values, which were interpreted automatically by RAPID@ IschemaView software and humanly from source workstation images for the same patients (human agreement reads), we saw excellent agreement (κ = 0.80; 95% CI, 0.60–0.90) for the dichotomized scores. In only one patient, the software underestimated the extent of early ischemic changes by providing an automated ASPECTS >6, while the score was <6 by agreement read.

### Cost Effectiveness Analysis (Figure 2)

The rate-adjusted total costs of the treatment decision based on automated interpretation and WhatsApp are summarized and extrapolated from our hospital cost (IV-tPA, cost of RAPID@IschemaView software, other stroke treatment and investigation, prolonged stay from complications, and death) in the Table 1.

**Figure 2:**
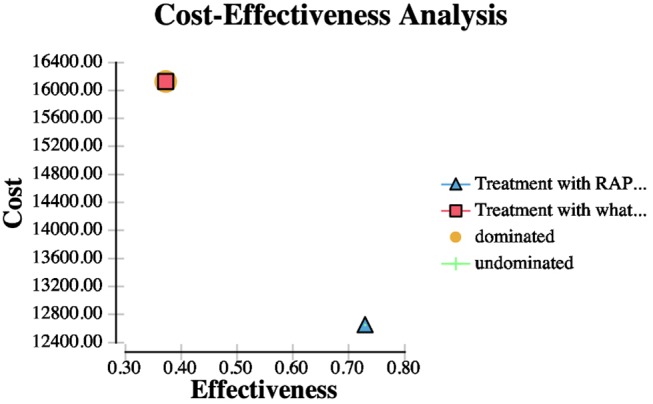
This figure demonstrates a CEA analysis curve for thrombolysis based on automated ASPECTS vs. ASPECTS interpretation on WhatsApp.

The use of WhatsApp to assess the ASPECTS had a total mean per patient cost of LE 16,126.48, and 0.37 QALYs, while automated ASPECTS had a total mean per patient cost of LE 12,646.48, and 0.73 QALYs. The incremental cost of WhatsApp supported ASPECTS interpretation over automated ASPECTS interpretation was LE 3,480.00. The estimated ICER for RAPID assessment vs. WhatsApp assessment of treatment decision was LE 9,738.35.

## Discussion

If regions of hypodensity encompass more than one-third of the affected cerebral hemisphere, IV thrombolysis is contraindicated and should not be administered (12). A *post-hoc* analysis of the European Cooperative Acute Stroke Study (ECASS) suggested that the extent of hypoattenuation on head CT was a predictor of response to thrombolysis and the risk of hemorrhagic transformation (13).

In early NNCT of AIS, judging the extent and degree of ischemia compared to infarction is not always straightforward. Even among experienced neuroradiologists and neurologists, the interrater agreement to determine whether ischemia affects less than or greater than one-third of the middle cerebral artery (MCA) territory is only moderate (κ.4) (14).

This draws attention to the effect of a quantitative approach in judging the extent of ischemia, and this includes the use of ASPECTS and the possibility for it to be integrated into the decision-making process for reperfusion therapy in patients with AIS, similarly to its use in deciding MT in LVO stroke guidelines (15). The current study reaffirmed the concern of interobserver variability for ASPECTS assessment by showing only fair interobserver agreement (κ = 0.57), even for experienced neuroradiologists. This was attributed to factors such as physician training and experience, time pressure, personal bias of expected findings (for example, from the ordering or treatment teams), and other factors that have been noted as potential reasons for the variability of ASPECTS (16–18). In the current study, this agreement severely dropped (κ = 0.36) when the assessment was for images sent on WhatsApp; this may be explained by the shortcomings of the image quality, which could affect the interpretation ability of that images. Marginal improvement in interobserver agreement (κ = 0.69) has been shown in our study when dichotomized ASPECTS (≥6 or <6) was used in agreement with other studies (19, 20). This Human variability in ASPECTS assessment could be the reason behind the contradiction between studies in linking ASPECTS assessment to predicting the clinical outcome (21, 22). In current studies excluding large core ischemia (hypoattenuation in >1/3 of MCA territory quantitatively equal to ASEPCTS <6), receiving IV-thrombolysis increased odds of functional independence and decreased odds of hemorrhagic complications in the more reliably selected patients based on automated ASPECTS interpretation of the early CT, which is similar to results of a study by Pfaff et al. (21). This improvement in reliability through the use of automated software has been shown in different studies, and the success of such software was achieved by addressing variability associated with human interpretation through software packages trained on deep learning algorithms (18, 21, 23–26). As shown by Maegerlein et al. (18), in the current study, there was excellent agreement (κ = 0.80) between dichotomized ASPECTS interpreted by RAPID and in the agreement reading of ASPECTS from source images on a workstation. The assessment of captured CT images on smartphones has several disadvantages that might hamper accurate interpretation of them. The quality of capturing files from a workstation onto a smartphone might be unfavorably affected by screen reflection from the workstation's monitor, lack of image stabilization, and the occasional loss of focus. Compared with a workstation, a smartphone has a smaller screen size, and image quality and resolution are obviously inferior. Standard, commonly used features available on a PACS for evaluating a CT image, such as manipulating the image by zooming in and out and changing the window level between soft tissue and bone, are not available when viewing a captured CT images on a smartphone. A CT image shown on a PACS can be readily scrolled through. Scrolling through captured CT images on a smartphone, while possible, is cumbersome compared with scrolling on a PACS. This may explain the discrepancy between neuroradiologists when they interpreted the ASPECTS value from source images on a workstation and from captured and sent images on WhatsApp. Consequently it is plausible to use automated ASPECTS to standardize NCCT interpretation in the acute setting, avoiding variability associated with individual human interpretation and ensuring that all patients receive equivalent care and are triaged with appropriate treatment options like in other studies (27).

In 2020, over 2 billion WhatsApp [a freeware, cross-platform messaging, and Voiceover IP (VoIP) service] users were reported worldwide. It has become the primary means of instant messaging in clinical and non-clinical settings in many countries and specialties. Its frequent use in stroke workflow could be due to its live-chat feature, i.e., instantaneous communication with all stroke team with transmitting and sharing a patient's CT images and clinical data with a real-time notification service. Remote viewing images on smartphones and tablets with specialized applications have been shown to be effective for rapidly visualizing radiologic images and for urgent decision making with regards to patient care in different domains of medicine (28, 29). The US Food and Drug Administration (FDA) has given multiple indications to the RAPID^@^IschemaView neuroimaging platform for the use of selecting stroke patients most likely to benefit from endovascular thrombectomy (30). However, these applications may appear to be costly and cumbersome because they require a prearranged setup, such as cloud computing or a visualization server at the source, and the installation and registration of the application at the receiving ends' smartphone. In the current study, a cost-effective analysis showed the use of rapid software with high reliability in screening patients presenting with acute stroke to determine eligibility for alteplase treatment is cost effective and warrants consideration as an alternative to routine practice compared to that using WhatsApp, which is a cheaper way of communication but is not cost effective and conducive to better healthcare. Like in several reports that support a favorable association between higher ASPECTS and good functional outcome (31, 32), we showed the use of an automated ASPECTS group [in group 1: median 7 (IQR, 6–8)] to exclude those patients with hypoattenuation in >1/3 of MCA territory (which quantitatively equal to ASEPCTS <6) was predictive of functional outcome. In the current study, there was no statistical difference between both groups of patients with regards to dichotomized ASPECTS score (ASPECTS ≥ 6/<6) in the agreement session (with improved k), neither with regards to initial median NIHSS or the basic clinical data, which make this discrepancy in outcome might ought to the higher reliability of automated ASPECTS (by RAPID@IschemaVeiw, used in current study) in interpreting ASPECTS for thrombolysis decision. That results which could not be affirmed in others studies were dated before the era of automated ASPECTS to include patients for thrombolysis (16, 33). There are several limitations to our study. The sample size was relatively small, and further validation studies with larger sample sizes are required to validate the practical application of our automated software as a stand-alone tool in the triage of patients with AIS for thrombolysis. A retrospective design can introduce unknown bias. Another issue is that no standardization was required for the quality of images captured for CT and shared on WhatsApp, but this could be of value to the current study to document the traditional style of communicating AIS workflow through a WhatsApp chat.

## Conclusion

We showed that interpretation of automated ASPECTS by the RAPID@IschemaView ASPECTS software package performs equally well with the agreement read of expert neuroradiologists, and this performance was severely degraded when WhatsApp captured CT images used for ASPECTS assessment. In our study, we showed that automated ASPECTS might predict outcomes following IV thrombolysis.

## Data Availability Statement

The datasets analyzed in this article are not publicly available. Requests to access the datasets should be directed to yassinossama@yahoo.com.

## Ethics Statement

The studies involving human participants were reviewed and approved by the Alexandria University Ethical Committee. Written informed consent for participation was not required for this study in accordance with the national legislation and the institutional requirements.

## Author Contributions

All authors contributed equally to drafting the work and revising it critically for important intellectual content. OM has substantial contributions to the conception and design of the work and the acquisition, analysis, and interpretation of data for the work, and contributed to drafting the work, revising it critically for important intellectual content. Final approval of the version to be published.

## Conflict of Interest

The authors declare that the research was conducted in the absence of any commercial or financial relationships that could be construed as a potential conflict of interest.

## Abbreviations

AIS: acute ischemic stroke
IQR: interquartile range
IV-tPA: Intravenous tissue plasminogen activator.
